# Book Reviews

**Published:** 1901-03

**Authors:** 


### *BOOK REVIEWS, PAMPHLETS, EXCHANGES.*

### *BOOK REVIEWS.*

[Contributions solicited for review.]

LESSONS ON THE ANATOMY, PHYSIOLOGY, AND HYGIENE OF INFANCY AND CHILDHOOD. Consisting of extracts from lectures given at Rush Medical College. By Alfred C. Cotton, A.M., M.D., Professor of Diseases of Children; Accoucheur and Physician for Diseases of Children, Presbyterian Hospital; Staff Member of the Central Free Dispensary and the Cook County Hospital; President of the Chicago Pediatric Society; Member of the American Pediatric Society, etc. One hundred illustrations. Cloth, $1.50 net, postpaid. Chicago Medical Book Co., Chicago, Ill.

This book is an acceptable presentation of the facts in this important branch. The author's long experience in pediatric practice will give the book additional weight.

---

A TEXT-BOOK ON PRACTICAL OBSTETRICS. By Egbert H. Grandin, M.D., and Geo. W. Jarman, M.D. Philadelphia. F. A. Davis Co. Third edition, revised and enlarged. Price, extra cloth, $4; sheep, $4.75.

This work presents a thorough review of the subject without unnecessary profuseness. In disputed points the authors have given that which was correct, in their judgment. The illustrations are numerous, mostly original, and add much to the value of the book. The book is well written, simple and forceful, and hence, readily understood.

---

A SYSTEM OF PRACTICAL THERAPEUTICS. Edited by Hobart A. Hare, M.D. Second edition. Revised and largely rewritten. Lea Bros. & Co. Philadelphia and New York. 1901.

The first of the volume deals with general therapeutic considerations, prescription writing, remedial measures other than drugs, diathetic diseases of nutrition. The work is based upon the system of therapeutics published ten years ago, but has naturally undergone great revision and amplification. The collaborators are taken from men well equipped to speak authoritatively on their given subjects. The work is destined to be one of the notable publications of the year.

---

THE AMERICAN YEAR BOOK OF MEDICINE AND SURGERY. Under the editorial charge of George M. Gould, M.D., Philadelphia and London. W. B. Saunders & Co. 1901.

The two volumes of the Year Book, one devoted to medicine, the other to surgery for 1901, show the same care in arrangement which have marked former issues of this valuable work. A full corps of collaborators have united in collecting the facts brought out in the progress of medicine during the past year, and this enables the reader to be placed fairly up to date. As a book of reference this cannot be excelled. The volumes may be purchased separately.

---

PHYSICAL DIAGNOSIS IN OBSTETRICS. By Edward A. Ayers, M.D., New York. E. B. Treat & Co. Price $2.

This work is a complete guide to diagnosis in ante-partum, partum and post-partum examinations. This is a subject that needs elucidation, and the book goes far toward accomplishing this end. It is fully and accurately illustrated.

